# Assessing and avoiding C isotopic contamination artefacts in mesocosm-scale ^13^CO_2_/^12^CO_2_ labelling systems: from biomass components to purified carbohydrates and dark respiration

**DOI:** 10.1186/s13007-025-01431-3

**Published:** 2025-08-11

**Authors:** Jianjun Zhu, Regina T. Hirl, Juan C. Baca Cabrera, Rudi Schäufele, Hans Schnyder

**Affiliations:** 1 Technische Universität München, Lehrstuhl für Grünlandlehre, Alte Akademie 12, 85354 Freising-Weihenstephan, Germany; 2 State Key Laboratory of Microbial Technology, Shandong University, Qingdao, Shandong China; 3 Institute of Bio- and Geoscience, Forschungszentrum Jülich GmbH, Agrosphere (IBG-3), Jülich, Germany; 4 Crop Physiology, Technische Universität München, 85354 Freising-Weihenstephan, Germany

**Keywords:** Atmospheric CO_2_ concentration, Bulk carbon, ^13^C isotopic labelling, ^13^C discrimination, Isotopic fractionation, C tracer, CO_2_ gas exchange, Contamination, Experimental artifact, Water-soluble carbohydrates (fructan, sucrose, glucose, fructose)

## Abstract

**Background:**

Quantitative understanding of plant carbon (C) metabolism by ^13^CO_2_/^12^CO_2_-labelling studies requires absence (or knowledge) of C-isotopic contamination artefacts during tracer application and sample processing. Surprisingly, this concern has not been addressed systematically and comprehensively yet is especially crucial in experiments at different atmospheric CO_2_ concentrations ([CO_2_]), when experimental protocols require frequent access to the labelling chambers. Here, we used a plant growth chamber-based ^13^CO_2_/^12^CO_2_ gas exchange-facility to address this topic. The facility comprised four independent units, with two chambers routinely operated in parallel under identical conditions except for the isotopic composition of CO_2_ supplied to them (δ^13^C_CO2_ −43.5‰ *versus* −5.6‰). In this setup, *d*δ^13^C_X_ (the measurements-based δ^13^C-difference between matching samples *X* collected from the parallel chambers) is expected to equal *d*δ^13^C_Ref_ (the predictable, non-contaminated δ^13^C-difference ), if sample-C is completely derived from the contrasting CO_2_ sources. Accordingly, contamination (*f*_contam_) was determined as *f*_contam_ = 1– *d*δ^13^C_X_/*d*δ^13^C_Ref_ in this experimental setup. Determinations were made for biomass fractions, water-soluble carbohydrate (WSC) components and dark respiration of *Lolium perenne* (perennial ryegrass) stands following growth for ∼9 weeks at 200, 400 or 800 µmol mol^− 1^ CO_2_, with a terminal two weeks-long period of extensive experimental disturbance of the chambers.

**Results:**

Contamination was small and similar (average 3.3% ±0.9% SD, *n* = 18) for shoot and root biomass and WSC fractions (fructan, sucrose, glucose, fructose) at every [CO_2_] level. [CO_2_] had no significant effect on contamination of these samples. There was no evidence for any contamination of WSC components during extraction, separation and analysis. At 200 and 400 µmol mol^− 1^ CO_2_, contamination of respiratory CO_2_ was close to that of biomass- and WSC-C, suggesting it originated primarily from in vivo-contaminated respiratory substrate. Surprisingly, we found no evidence of contamination of respiratory CO_2_ at 800 µmol mol^− 1^ CO_2_. Overall, contamination likely resulted overwhelmingly from photosynthetic fixation of extraneous contaminating CO_2_ which entered chambers primarily during daytime experimental activities.

**Conclusions:**

The labelling facility enables months-long, quantitative ^13^CO_2_/^12^CO_2_-labelling of large numbers of plants with accuracy and precision across contrasts of [CO_2_], empowering eco-physiological study of climate change scenarios. Effective protocols for contamination avoidance are discussed.

**Supplementary Information:**

The online version contains supplementary material available at 10.1186/s13007-025-01431-3.

## Background

Isotopic labelling of the carbon (C) in CO_2_ supplied to photosynthesizing organisms is a unique and powerful method for investigating C fluxes in central metabolism, transport, allocation and partitioning of photosynthetic products from the organelle (chloroplast) to the ecosystem scale [1–17]. Multiple different techniques, including pulse-chase and dynamic labelling (*sensu* Ratcliffe & Shachar-Hill [18]; or synonymous ‘steady-state’ or ‘continuous’ labelling [7]) with different C isotopes (^11^C, ^13^C or ^14^C) have been designed and applied to different aspects of the analysis of C fluxes in plants [6, 7, 19–22]. One such method is especially useful for long-term (hours- to months-long) labelling at large scales, with large numbers of plants in controlled environments, and uses inexpensive and harmless near-natural abundance ^13^CO_2_/^12^CO_2_ mixtures [21, 23–25]. These are derived from ^13^C-depleted fossil-organic or (relatively) ^13^C-enriched mineral sources and thus termed ‘fossil-organic’ or ‘mineral CO_2_’. This technique has proven useful for the determination of functional components of CO_2_ fluxes, such as dark respiration in light [25, 26], distinction of autotrophic and heterotrophic ecosystem respiration [21] and quantification of the labelling kinetics of metabolic and storage substrate pools supplying sink tissue [27, 28] or dark respiration of shoots and roots [29, 30]. Further, such tracer studies have enabled analysis of C fluxes in central carbohydrate metabolism of source leaves and of the function and importance of assimilate stores (or reserves) in supplying substrate to growth or respiration by compartmental models at organ, plant and ecosystem scale [27, 31–33].

**Fig. 1:**
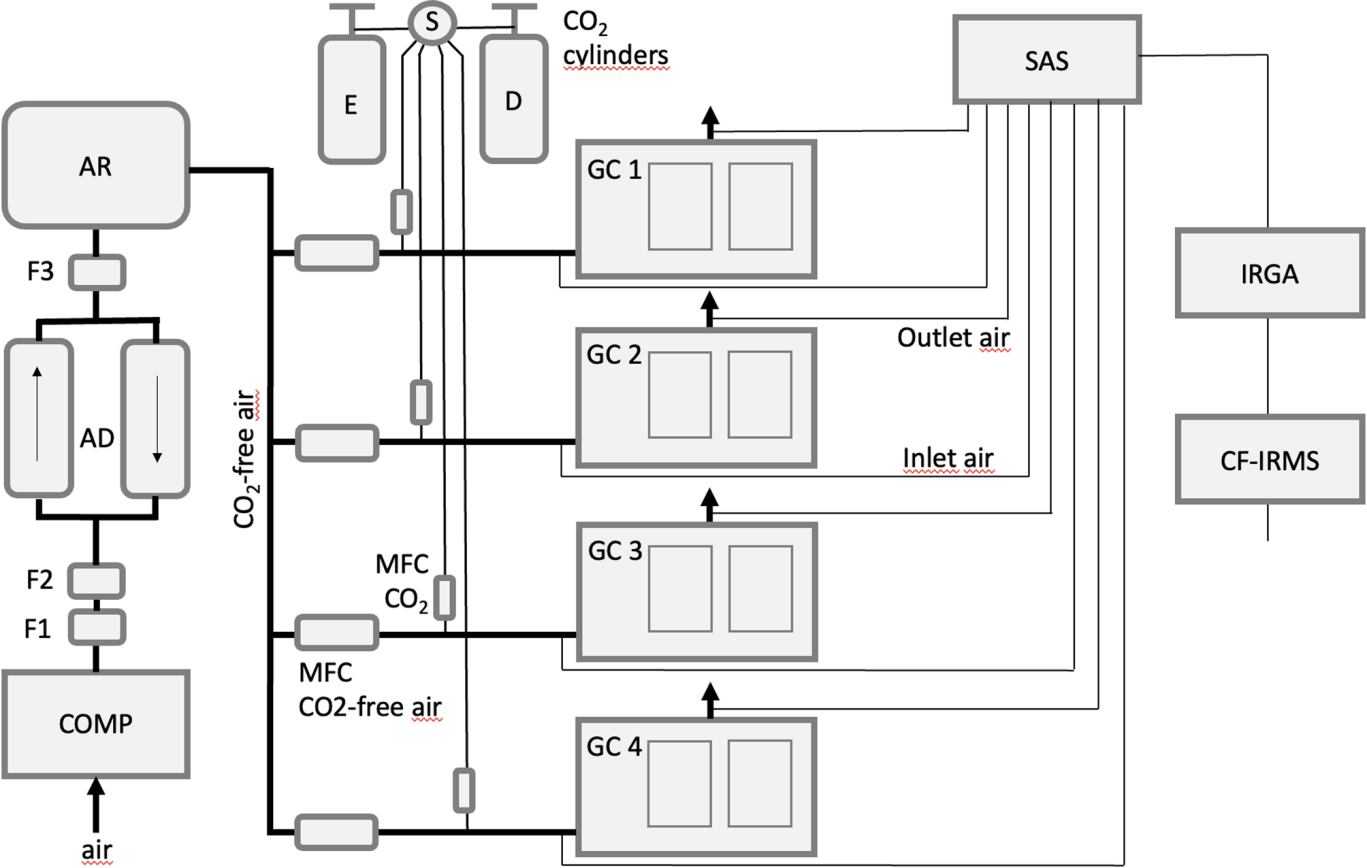
Schematic diagram of the ^13^CO_2_/^12^CO_2_ labelling and gas exchange system. COMP, screw compressor (S40, Boge, Bielefeld, Germany); F1, oil and water condensate drain (CSP005; Hiross, Mönchengladbach, Germany); F2, oil, water and particle filter (≥ 0.01 μm; G12XD, with filter element 2030X, Zander); AD, adsorption dryer (KEN 3100 TE; Zander, Essen, Germany) and molecular sieve: activated aluminium oxide F200; Alcoa, Houston, TX, USA); F3, universal filter (≥ 1 μm; G12ZHD and filter element: 2030Z, Zander); AR, air receiver (1 m^3^) (Magnet Kft, Magocs, Hungary); E and D, cylinders with ^13^C-depleted (fossil) and -enriched (mineral) CO_2_ from Linde AG (Unterschleissheim, Germany) and CARBO Kohlensäurewerke (Bad Hönningen, Germany); S, CO_2_ source unit for mineral and fossil-organic CO_2_ (DMP Ltd, Fehraltdorf, Switzerland); MFC CO_2_, CO_2_ mass flow controller (Red-y, Vögtlin, Muttenz, Switzerland, max 1 SLPM); MFC air, mass flow controller for CO_2_ free air (EL-FLOW, Bronkhorst, Veenendaal, Netherlands; 1000 SLPM); GC 1–4, growth chambers (PGR15; Conviron, Winnipeg, Canada); SAS, sample air selector (DMP Ltd, Fehraltdorf, Switzerland); IRGA, CO_2_ and H_2_O infrared gas analyser (Li-840, Li-Cor Inc., Lincoln, NE, USA); CF-IRMS, continuous-flow ^13^CO_2_/^12^CO_2_ isotope ratio mass spectrometer (Delta plus; Finnigan MAT, Bremen, Germany). For simplicity, a number of auxillary components of the facility are not included in the figure (but see text)

A special variant of this labelling strategy– particularly useful for systematic and comprehensive exploration of common contamination artefacts (as we show here)– uses two parallel identical growth chambers with the same plant material grown in the same conditions except for the C isotopic composition (δ^13^C, Table 1) of the CO_2_ (δ^13^C_CO2_) supplied to the chambers. In our laboratory, such a system is directly connected with a continuous-flow stable isotope ratio mass spectrometer (CF-IRMS) which permits quasi-continuous monitoring of δ^13^C_CO2_ at the chamber inlet and outlet of the air stream passing through the chambers (Fig. 1). As air is ventilated strongly inside the chambers, the δ^13^C_CO2_ at the chamber outlet reflects that inside the chamber [25] as in leaf cuvettes [34].

In the field, as well as in open experimental systems (such as flow-through leaf cuvettes or mesocosms, as here), the δ^13^C of plant biomass is generally ^13^C-depleted relative to CO_2_ because of ^13^C discrimination (Δ^13^C), i.e. isotopic fractionation against ^13^C, in photosynthesis [35, 36], possibly modified further to a smaller degree by post-photosynthetic isotopic fractionation effects [29, 37–40]. According to Farquhar et al. [35, 36], δ^13^C of a given plant sample *X* (tissue or compound) is related to δ^13^C_CO2_ as.

1 $${{\delta\:}}^{13}{\text{C}}_{\text{X}}\:=\:({{\delta\:}}^{13}{\text{C}}_{\text{C}\text{O}2}\:\--\:{\Delta}^{13}{\text{C}}_{\text{X}})\:/\:(1\:+\:{\Delta}^{13}{\text{C}}_{\text{X}}),$$

with Δ^13^C_X_ representing the sample-specific Δ^13^C (which integrates both photosynthetic and eventual post-photosynthetic effects). Although Δ^13^C_X_ can vary as a function of environmental conditions [41, 42], it is theoretically independent of the isotopic composition of CO_2_ [36] and, hence, must be the same when plants are grown in identical conditions with different δ^13^C_CO2_ [24, 25].

**Table 1:** Definition of symbols, and specifications

Symbol	Definition	Specification
δ^13^C	Defined as δ^13^C = (*R*_P_/*R*_S_– 1) × 1000, with *R* the molar abundance ratio ^13^C/^12^C, and *P* referring to the sample and *S* to the international Vienna-Pee Dee Belemnite (V-PDB) standard (‰)	Farquhar et al. [36]
δ^13^C_CO2_	δ^13^C of CO_2_ (‰)	Here we used CO_2_ of mineral (δ^13^C_CO2_ ~–5.6‰) and fossil-organic origin (δ^13^C_CO2_ ~–43.5‰) to supply parallel growth chambers
δ^13^C_inlet_	δ^13^C of CO_2_ at the inlet of a growth chamber (‰)	Measured
δ^13^C_outlet_	δ^13^C of CO_2_ at the outlet of a growth chamber (‰)	Measured
δ^13^C_outlet pure_	δ^13^C of uncontaminated CO_2_ at the outlet of a growth chamber (‰)	Calculated as: δ^13^C_outlet pure_ = (Δ^13^C + ξ δ^13^C_inlet_ Δ^13^C/1000 + ξ δ^13^C_inlet_)/( Δ^13^C/1000 ( ξ– 1) + ξ), with Δ^13^C fixed at 21‰
δ^13^C_X_	δ^13^C of sample *X* (‰), with *X* referring to net photosynthesis, dark respiration, biomass, or WSC in the form of fructan, sucrose, glucose or fructose	Measured
δ^13^C_WSC_	δ^13^C of water-soluble carbohydrates (WSC) (‰)	Measured
δ^13^C_WSC−free biomass_	δ^13^C of WSC-free biomass (‰)	Calculated as δ^13^C_WSC−free biomass_ = (δ^13^C_biomass_ × *W*_biomass_– δ^13^C_WSC_ × *W*_WSC_)/(*W*_biomass_– *W*_WSC_)
*d*δ^13^C_X_	δ^13^C-difference between samples of the same kind (net photosynthesis, dark respiration, biomass, or WSC, in the form of fructan, sucrose, glucose or fructose) collected simultaneously from parallel chambers supplied with CO_2_ of contrasting δ^13^C_CO2_ (‰)	Based on measurements
δ^13^C_Ref_	δ^13^C of uncontaminated (pure) reference (‰)	Calculated as δ^13^C_Ref_ = (δ^13^C_inlet_ × *F*_inlet_– δ^13^C_outlet pure_ × *F*_outlet_) / (*F*_inlet_– *F*_outlet_)
*d*δ^13^C_Ref_	δ^13^C-difference between uncontaminated (pure) references from parallel chambers supplied with CO_2_ of contrasting δ^13^C_CO2_ (‰)	Based on calculations of δ^13^C_Ref_ for ‘samples’ collected simultaneously from parallel chambers supplied with contrasting δ^13^C_CO2_
*f*_contam X_	Fraction of contaminating C in sample *X*	Calculated as 1– *d*δ^13^C_X_ /dδ^13^C_Ref_
Δ^13^C	Carbon isotope discrimination (‰)	Farquhar et al. [36], here set to 21‰ in estimations of δ^13^C_Ref_
Δ^13^C_X_	Carbon isotope discrimination as expressed in sample *X* (‰)	Based on measurements, and calculated as Δ^13^C_X_ = (δ^13^C_outlet_– δ^13^C_X_)/(1 + δ^13^C_X_/1000)
ξ	Ratio of the rate of CO_2_ entry into a growth chamber relative to the net rate of CO_2_ uptake (net photosynthesis)	After Evans et al. [34] Calculated as ξ = *C*_inlet_ */* (*C*_inlet_– *C*_outlet_)
[CO_2_]	CO_2_ concentration in air (µmol mol^− 1^)	
*C*_inlet_	CO_2_ concentration in air at the inlet of the growth chamber (µmol mol^− 1^)	Measured
*C*_outlet_	CO_2_ concentration in air at the outlet of the growth chamber (µmol mol^− 1^)	Measured
*F*_inlet_	Flux of CO_2_ entering a growth chamber (µmol s^− 1^)	Based on measurements
*F*_outlet_	Flux of CO_2_ leaving a growth chamber (µmol s^− 1^)	Based on measurements
*A*	Ground area of a growth chamber (m^2^)	
*N*	Net CO_2_ exchange rate in light, i.e. whole-stand net photosynthesis rate (µmol m^− 2^ s^− 1^)	*N* = (*F*_inlet_– *F*_outlet_) / *A*, during daytime
*R*_n_	Whole-stand respiration rate in the dark (µmol m^− 2^ s^− 1^)	*R*_n_ = (*F*_inlet_– *F*_outlet_) / *A*, during nighttime
*W*_biomass_	C mass of a certain biomass sample (g)	Measured
*W*_WSC_	C mass of WSC in a certain sample (g)	Based on measurements and the mass fraction of C in different forms of water-soluble carbohydrates (fructan ~ 0.44, sucrose 0.42, glucose and fructose 0.40)
*X*	Designation of samples of a given kind collected simultaneously from parallel chambers supplied with contrasting CO_2_; may refer to dark respiration, biomass, or WSC (fructan, sucrose, glucose, fructose)	Here, CO_2_ of mineral (δ^13^C_CO2_ ~ − 5.6‰) or fossil-organic (δ^13^C_CO2_ ~–43.5‰) origin

Therefore, when established in the above two-chamber system, the δ^13^C of an uncontaminated (pure) plant C sample (termed δ^13^C_Ref_) which is synthesized completely from photosynthetic CO_2_ uptake of a certain CO_2_ source is expected to accord with Eq. 1 independently of the δ^13^C_CO2_ of the source CO_2_. Accordingly– and again in artefact-free conditions and steady-state– the δ^13^C-difference (*d*δ^13^C_Ref_) between chambers supplied with ^13^C-enriched (mineral) and ^13^C-depleted (fossil) CO_2_ should be identical to that predicted using Eq. 1. Any C contamination of an actual sample *X* would cause a (contamination-weighted) decrease of *d*δ^13^C_X actual_ relative to *d*δ^13^C_Ref_ (i.e. *d*δ^13^C_X actual_ < *d*δ^13^C_Ref_). In the extreme, where *d*δ^13^C_X_ = 0, the sample *X* is fully independent of the different δ^13^C_CO2_ used, i.e. is completely contaminated. Accordingly, the fraction of contaminating C in a certain sample *X* (*f*_contam X_) can be defined as:

2 $${f}_{\text{c}\text{o}\text{n}\text{t}\text{a}\text{m}\:\text{X}}\:=\:1\:\--\:d{{\delta\:}}^{13}{\text{C}}_{\text{X}\:\text{a}\text{c}\text{t}\text{u}\text{a}\text{l}}\:\:/\:d{{\delta\:}}^{13}{\text{C}}_{\text{R}\text{e}\text{f}}.$$

*Ceteris paribus*, a given contaminating C source has the same δ^13^C and adds the same quantity of C to a certain sample *X* collected from the parallel chambers which are fed with different δ^13^C_CO2_. This is true especially, if the parallel chambers are operated simultaneously, are housed in the same room, and sample collection and processing use identical protocols (as was the case in this work). Putative contaminating C sources are many and include (1) free atmospheric CO_2_ (which has a δ^13^C of approx. − 9‰ at present [43]), (2) CO_2_ exhaled by people (e.g. experimenters; − 17 and − 25‰ [44, 45]), and (3) cross-contamination with the different labelling CO_2_s [24, 25]. Further, (4) contamination with organic C compounds might occur during sample collection or processing [46, 47]. In the context, also (5) seed biomass-C (or biomass of any type of experimental starting material, e.g. vegetative cuttings or seedlings) ‘qualifies’ as a contaminant, as it shares the same δ^13^C in the different labelling chambers. Particularly, in climate change experiments, the likelihood and extent of contamination could perhaps depend on the atmospheric CO_2_ concentration, [CO_2_], which is used in the experiments. This would cause a [CO_2_]-dependent experimental artefact and bias conclusions, if unnoted or uncorrected. As far as we know, there have been no systematic, comprehensive analyses of contamination artefacts in large- or stand-scale, long-term C labelling studies (but see Gong et al. [26]). Particularly, we know of no such methodological study under sub-ambient or elevated [CO_2_] conditions.

In this work, we ask: How does [CO_2_] affect C contamination (*f*_contam_) of a range of parameters that are of interest in labelling studies, including biomass fractions (shoot and root), non-structural carbohydrate components (water-soluble carbohydrates (WSC): fructan, sucrose, glucose, fructose) and dark respiration? In addition, we perform a sensitivity analysis of C isotope discrimination (Δ^13^C) assumptions on the estimates of contamination. At the outset, we provide a description of the custom-made labelling facility used here. The work was performed with stands of *Lolium perenne* (perennial ryegrass, C_3_) established from 12 days-old seedlings grown in parallel growth chambers under identical conditions with contrasting δ^13^C_CO2_ (i.e. δ^13^C_CO2_ of −43.5‰ or −5.6‰) at 200, 400 or 800 µmol mol^− 1^ CO_2_, approximating Last Glacial Maximum, current ambient, or predicted end-of-the 21st century [CO_2_] levels [48]. Biomass samples for contamination analysis were collected immediately after the terminal, two weeks-long experimental period in which the labelling vessels (growth chambers) had to be accessed frequently for plant sampling or non-destructive measurements [48–50]. These perturbations provided a special opportunity for contamination of the chamber atmospheres with extraneous CO_2_.

## Materials and methods

### Mesocosm-scale ^13^CO_2_/^12^CO_2_ gas exchange and labelling system

The ^13^CO_2_/^12^CO_2_ gas exchange and labelling facility corresponded to a modernized and upgraded version of the system originally described by Schnyder et al. [25]. The facility was composed of four main modules (Figs. 1, S1 and S2): (1) a screw compressor and adsorption dryer which generated CO_2_-free air, (2) a gas mixing system which controlled the addition of CO_2_ to CO_2_-free air and supplied air with known δ^13^C_CO2_ and [CO_2_] at an individually set rate for both air flow and [CO_2_] to each labelling vessel, (3) four plant growth chambers, which served as the labelling vessels, and (4) a gas analysis unit, comprising a sample air selector, an infrared CO_2_ gas analyzer (IRGA) and a continuous flow ^13^CO_2_/^12^CO_2_ IRMS, which analyzed in sequence the [CO_2_] and δ^13^C_CO2_ of sample gas collected at the inlet and outlet of each chamber.

Specifically, the four growth chambers served as open-system [51], mesocosm-scale gas exchange cuvettes, each having a 1.5 m^2^ plant growth area and equipped with a microprocessor controller and environmental data acquisition system. All air supply to a growth chamber was provided by a dedicated gas mixing station which consisted of two computer-controlled mass flow controllers (Fig. 1) which regulated the mixing of CO_2_ with known δ^13^C (0–1 standard liter per minute, SLPM) and CO_2_-free air (0–1000 SLPM). Dry CO_2_-free air was obtained with a self-regenerating adsorption dryer at up to 180 m^3^ h^− 1^ at ambient atmospheric pressure. The dryer was fed with compressed air (approx. 7 MPa) by a screw compressor *via* an oil and water condensate drain and filters as shown in Fig. 1. Commercially available CO_2_ of known δ^13^C was supplied from cylinders (Fig. 1). Typically, rates of air supply to individual chambers ranged between 250 and 750 SLPM. Thus, with an internal chamber volume of approx. 3000 L, air flow through a chamber was equal to 5–15 times the chamber volume per hour. Accordingly, the mean residence time of CO_2_ in the chamber was 4–12 min. Sample air was collected at the inlet and outlet of each growth chamber and continuously pumped to the computer-controlled sample air selector (SAS) at a rate of approx. 2 L min^− 1^. During simultaneous operation of all chambers the SAS sequentially sampled each sample air line (*n* = 8; Fig. 1) at approx. 3 minutes-intervals. Sample air was split to serve the IRGA and CF-IRMS in parallel. Gas lines between the SAS and CF-IRMS and IRGA were flushed with sample air for 3 min before taking IRGA readings of CO_2_ and H_2_O concentration and measurement of δ^13^C by the CF-IRMS. The CF-IRMS was interfaced with the sample air selector *via* a steel capillary tube (1 mm i.d.), a eight-port, two-position valve (Valco Instruments Co. Inc., Houston, TX, USA), dryer (Nafion^®^), gas chromatograph (25 m × 0.32 mm Poraplot Q; Chrompack, Middelburg, Netherlands) and open split. These components all formed part of a custom-made interface (Gasbench II; ThermoFinnigan, Bremen, Germany). Sample air for the CF-IRMS was pumped continuously through the steel capillary feeding the Valco valve and a 0.25 mL sample loop attached to it. After a 90 s flushing period, the content of the sample loop was swept with helium carrier gas through the interface, where water vapor was removed by the Nafion trap and CO_2_ was separated from other sample air gases in a GC column. Finally, the CO_2_ was introduced directly into the ion source of the IRMS via a glass capillary (0.1 mm i.d.) connected to the interface by an open split. After another 90 s, shortly before the sample air selector switched to the next sample air line, a second sample of the same air was taken. Thus, within 3 min, each inlet/outlet was measured in duplicate. After every second sample, a VPDB-gauged CO_2_ reference gas was injected into the CF-IRMS *via* the open split. A full measurement cycle, including one set of measurements (concentrations of CO_2_ and H_2_O, and δ^13^C of CO_2_) on the inlet and outlet of each growth chamber, was completed within less than 30 min. The long-term precision (SD) for repeated measurements at the chamber inlet was < 0.20‰.

Empty chamber tests performed before every experiment confirmed that gas lines throughout the air supply systems of the chambers were virtually leak-free based on measurements with CO_2_ free air generated by the adsorption dryer and CF-IRMS based measurement of the peak size (observed peak area corresponded to a CO_2_ concentration of < < 0.5 µmol mol^− 1^) of mass 44, i.e. ^12^C^16^O_2,_ at the chamber inlet. The same was true for measurements at the chamber outlet, when the flow rate of CO_2_-free air was maintained at > 250 SLPM, as was routinely the case in experiments.

Individual CO_2_ cylinders contained approx. 30 kg of CO_2_, and more than one cylinder had to be used in experiments of long duration (> 4 weeks). For this reason, we examined batches of CO_2_ cylinders for uniformity of their δ^13^C_CO2_. Typically, the δ^13^C_CO2_ was quite similar (< 0.27‰ SD) between cylinders of the same type (mineral or fossil-organic CO_2_) within a batch.

### Plant material and growth conditions

The details for the plant material and growth conditions used in this study have been presented before [48–50]. In short, plants of *Lolium perenne* were established and grown singly in individual plastic pots (350 mm height, 50 mm diameter) filled with 800 g of washed quartz sand (0.3–0.8 mm grain size). Pots were arranged at a density of 383 plants m^− 2^ in plastic containers (770 × 560 × 300 mm), and two of such containers placed in each growth chamber. Plants were supplied four times a day with a Hoagland-type nutrient solution with reduced nitrate-N content [48]. Light was supplied by cool-white, fluorescent tubes and warm-white, light-emitting diode (LED) bulbs with a constant photosynthetic photon flux density (PPFD) of 800 µmol m^− 2^ s^− 1^ at plant height during the 16 h-long light period [48]. Temperature was controlled at 20 °C/16°C and relative humidity (RH) at 50%/75% during the light/dark periods. Importantly, we observed no chamber effects on any measured parameter in the studies of Baca Cabrera et al. [48–50].

### [CO_2_] treatments and sequence of experimental activities and sampling

[CO_2_] treatments were installed when seedlings were 12 days old following seed imbibition. In each of two experimental runs chamber air was controlled near the target CO_2_ concentration ([CO_2_] of 200, 400 or 800 µmol mol^− 1^) [48] with two chambers per [CO_2_] treatment. In that, one chamber was supplied with ^13^C-enriched (mineral) CO_2_ and the other with ^13^C-depleted (fossil-organic) CO_2_. Maintenance of [CO_2_] near target values throughout the experiment– from 12 days-old seedlings to closed stands and beyond– required periodic adjustments of airflow and [CO_2_] at the chamber inlet. This was done in such a way that the (photosynthetic) drawdown of [CO_2_] inside the chambers did not exceed 14%. Quasi-continuous ^13^CO_2_/^12^CO_2_ measurements at the inlet and outlet of chambers were performed from day 20 to at least day 65.

Disturbance of the [CO_2_] and δ^13^C_CO2_ in the chambers was minimized by maintaining a small overpressure in the chambers relative to the outside atmosphere (Figure S2D) and by restricting daytime experimental activities inside the chambers between days 49 and 63 as much as possible within the limitations of the experimental plan [48–50]. Also, air locks (Figure S3A) were installed in chamber doors throughout the 14 days-long period of active experimentation. For the latter chambers had to be routinely accessed daily before the end of the light period for (non-destructive) measurements of leaf elongation on eight plants per chamber [48]. In parallel, leaf level gas exchange measurements (not reported here) were made on individual plants [48]. These measurements were performed in a different, dedicated growth chamber which was controlled at the same [CO_2_] with the same δ^13^C_CO2_ as the chamber of origin of a given plant. Thus, individual plants were removed from their chambers for leaf level gas exchange measurements and later returned to their chamber of origin [48]. In addition, intensive sampling activities over two consecutive days occurred before the end of the light and dark periods on days 49 and 50, and days 63 and 64 (data not reported here, but partly presently in Baca Cabrera et al. [49, 50]).

**Fig. 2:**
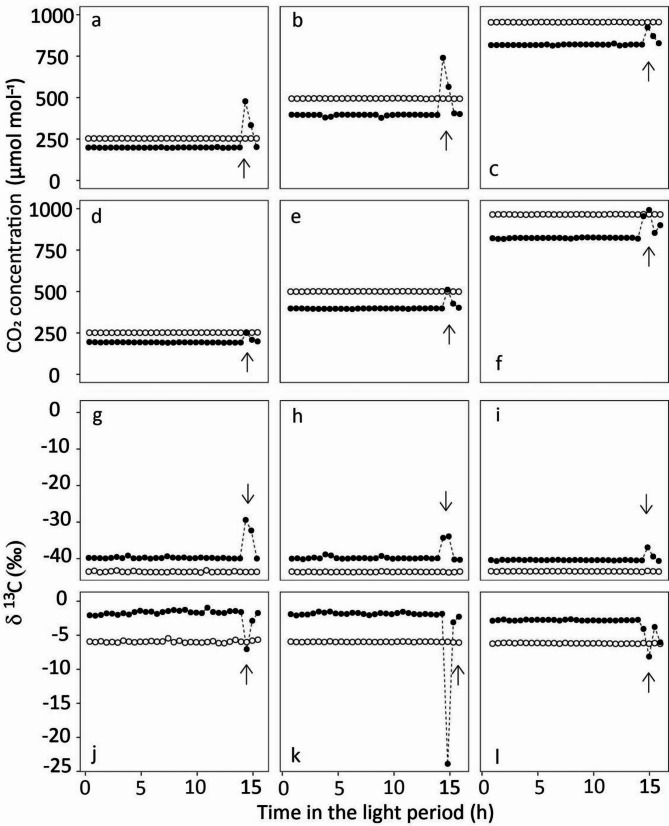
Concentration (a - f) and δ^13^C (g - l) of CO_2_ inside growth chambers during one light period. Growth chambers were maintained near target [CO_2_] of 200 (a, d, g and j), 400 (b, e, h and k), or 800 (c, f, i and l) µmol mol^− 1^ with either ^13^C-depleted CO_2_ (δ^13^C_CO2_ -43.5‰) ( a, b, c, g, h, and i) or ^13^C-enriched CO_2_ (δ^13^C_CO2_ -5.6‰; right) ( d, e, f, j, k, and l). Open circles denote measurements at the chamber outlet, and closed circles at the chamber inlet. Vertical arrows indicate chamber door openings during sampling activities on day 49. Data points represent individual measurements

The above activities intrinsically meant a disturbance which generated opportunities for contamination of the chamber atmospheres with extraneous CO_2_ (Fig. 2). Here, we quantify the cumulative effect of all putative sources of contamination (see Background) on the δ^13^C of plant biomass and WSC components. For this, we sampled plants shortly after the end of the intensive experimental period (day 65) at the beginning of the dark period. Two replicate samples from each growth chamber were collected, with one replicate consisting of three randomly selected plants. Plants were removed from their pot, their roots washed to free them of sand and dissected into their shoot and root parts. The plant parts were weighed to determine their fresh weight, then frozen in liquid nitrogen and stored at −18 °C before freeze-drying for 72 h. Dry weights were subsequently determined. After that, plant material was ground to a fine powder in a ball mill (Mixer mill MM 400, Retsch, Haan, Germany) in 2-mL stainless steel grinding jars with 0.5-mm stainless steel beads, and thereafter stored again at −18 °C until further use.

### WSC extraction and separation

WSC were extracted from shoot samples and fractions (fructan, sucrose, glucose, and fructose) separated using the procedures described by Gebbing & Schnyder [52]. Briefly, aliquots of 200 mg of milled sample material were weighed into 2-mL capped Eppendorf tubes and topped off with 1.8 mL of deionized water. Tubes were briefly vortexed (Vortex-Genie 2, Scientific Industries, New York, USA), held in a water bath at 93 °C for 10 min, shaken for 45 min (Shaker, Heidolph Instruments, Schwabach, Germany) at room temperature, and then centrifuged at 9500 *g* for 15 min (Universal 320, Merck, Tuttlingen, Germany). The supernatant, which contained the dissolved WSC, was passed through nylon-membrane filters with a pore size of 0.45 μm and then stored in clean 2-mL capped Eppendorf tubes at − 18 °C.

**Fig. 3:**
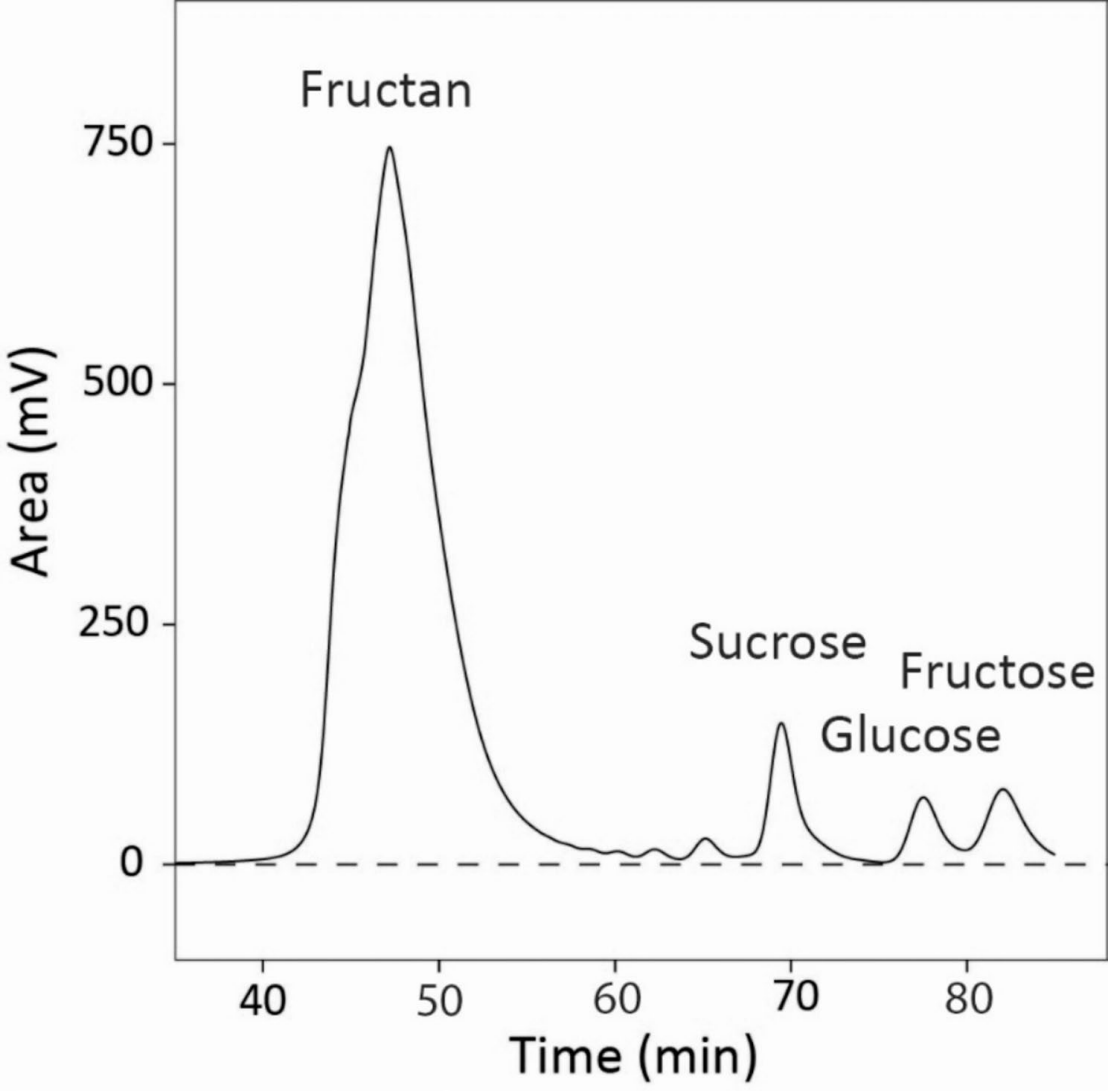
Typical HLPC elution diagram for water-soluble carbohydrates (WSC) extracted from whole shoot biomass of *Lolium perenne*. The fractions corresponding to fructan, sucrose, glucose and fructose are indicated in the panel. Note the two small peaks on the lefthand side of the sucrose peak, which likely corresponded (from right to left) to fructan tri-saccharides and tetra-saccharides. The thin grey line represents the baseline. The total elution time was about 90 min following sample injection. The sample was taken from plants grown in 800 µmol mol^− 1^ [CO_2_]

WSC fractions (fructan, sucrose, glucose and fructose) were separated, quantified and collected using a high-performance liquid chromatography (HPLC) system similar to that of Gebbing & Schnyder [52]. Thus, 0.2 mL aliquots of the filtered supernatant were passed through a guard column (Shodex KS-LG, Showa Denko, Tokyo, Japan) and a preparative column (Shodex Sugar KS2002, 300 × 20 mm, Showa Denko, Tokyo, Japan) held at 50 °C, with HPLC-grade water (Carl Roth, Karlsruhe, Germany) as the eluent, at a flow rate of 0.75 mL min^− 1^. The WSC were detected by refractive index measurement (Shodex RI-101, Showa Denko, Tokyo, Japan) and concentrations quantified by comparing sample peak areas against reference calibration curves of pure and mixed standards of analytical grade inulin, sucrose, glucose and fructose (all from Merck, Darmstadt, Germany). Knowing when the individual carbohydrates eluted from the preparative column (Fig. 3), fractions of fructan, sucrose, glucose, and fructose were individually collected in test tubes.

### ^13^C analysis of biomass and water-soluble carbohydrate components

The δ^13^C of biomass samples was determined for all shoot and root replicates, as in Lattanzi et al. [27]. The stored samples were thawed, re-dried at 40 °C for 24 h and stored in exsiccator vessels. Aliquots of 0.70 ± 0.05 mg of the shoot and root materials were weighed and packed into tin cups (3.3 × 5 mm, IVA Analysentechnik, Meerbusch, Germany). These were then combusted in an elemental analyzer (NA 1110, Carlo Erba Instruments, Milan, Italy) interfaced (Conflo III, Finnigan MAT, Bremen, Germany) to a continuous-flow isotope-ratio mass spectrometer (CF-IRMS, Delta Plus, Finnigan MAT, Bremen, Germany) which measured δ^13^C. A solid internal laboratory standard (SILS, fine ground wheat flour) was measured as a reference after every tenth sample to correct for possible instrument drift. All samples and SILS were measured against a laboratory working standard CO_2_ gas, which was previously calibrated against a secondary isotope standard (IAEA-CH6; calibration accuracy ± 0.06‰ SD). The long-term precision given as the SD of repeated measurements of the SILS was < 0.2‰.

Aliquots of approximately 0.70 mg of the different WSC fractions were transferred to tin cups, dried at 60 °C for 24 h, and then analyzed for their δ^13^C using the same CF-IRMS system as above.

### δ^13^C of WSC-free biomass

The δ^13^C of WSC free biomass (δ^13^C_WSC−free biomass_) was determined from isotopic mass balance for a given biomass sample *X*, thus.

3 $$\begin{array}{l}{{\delta\:}}^{13}{\text{C}}_{\text{W}\text{S}\text{C}-\text{f}\text{r}\text{e}\text{e}\:\text{b}\text{i}\text{o}\text{m}\text{a}\text{s}\text{s}}\:=\\\:({{\delta\:}}^{13}{\text{C}}_{\text{b}\text{i}\text{o}\text{m}\text{a}\text{s}\text{s}}\:\:{W}_{\text{b}\text{i}\text{o}\text{m}\text{a}\text{s}\text{s}}\:\--\\\:{{\delta\:}}^{13}{\text{C}}_{\text{W}\text{S}\text{C}}\:\:{W}_{\text{W}\text{S}\text{C}})\:/\:({W}_{\text{b}\text{i}\text{o}\text{m}\text{a}\text{s}\text{s}}\:\--\:{W}_{\text{W}\text{S}\text{C}}),\end{array}$$

with *W*_biomass_ and *W*_WSC_ the C mass in biomass and in total WSC of a give sample, and δ^13^C_biomass_ and δ^13^C_WSC_ the of δ^13^C of the biomass and WSC extracted from that biomass sample. Note that the isotopic mass balance accounts explicitly for isotope fractionation (^13^C discrimination) effects on bulk shoot biomass and WSC as expressed in their δ^13^C_biomass_ and δ^13^C_WSC_ (cf. Eq. 1).

### δ^13^C of respired CO_2_

The δ^13^C of respired CO_2_ (δ^13^C_Rn_) was obtained as [25]:

4 $$\begin{array}{l}{{\delta\:}}^{13}{\text{C}}_{\text{R}\text{n}}\:=\\\:({{\delta\:}}^{13}{\text{C}}_{\text{i}\text{n}\text{l}\text{e}\text{t}}\:\:{F}_{\text{i}\text{n}\text{l}\text{e}\text{t}}\:\--\:{{\delta\:}}^{13}{\text{C}}_{\text{o}\text{u}\text{t}\text{l}\text{e}\text{t}}\:\:{F}_{\text{o}\text{u}\text{t}\text{l}\text{e}\text{t}})\:/\:({F}_{\text{i}\text{n}\text{l}\text{e}\text{t}}\:\--\:{F}_{\text{o}\text{u}\text{t}\text{l}\text{e}\text{t}}),\end{array}$$

with δ^13^C_inlet_ and δ^13^C_outlet_ the (measured) δ^13^C of CO_2_ entering and leaving the growth chamber, respectively, and *F*_inlet_ and *F*_outlet_ the fluxes of CO_2_ (µmol s^− 1^) entering and leaving the chamber during the dark period.

### Estimation of C contamination

The fraction contamination of the C (*f*_contam_) contained in any one type *X* of sample (with *X* standing for biomass or WSC fraction (fructan, sucrose, glucose or fructose) or respired CO_2_ was determined as *f*_contam X_ = 1– *d*δ^13^C_X actual_/*d*δ^13^C_Ref_ (Eq. 2) as explained in the Background section. In this, *d*δ^13^C_X_ corresponds to the measurements-based δ^13^C-difference between samples of the same type collected simultaneously from parallel chambers, where one was supplied with ^13^C-depleted CO_2_ and the other with ^13^C-enriched CO_2_. Meanwhile, *d*δ^13^C_Ref_ refers to an estimation of the contamination-free δ^13^C-difference between the ^13^C-depleted and ^13^C-enriched CO_2_ supplied to the chambers for the reference sample (see below and Table 1). For calculation of δ^13^C_Ref_ for each chamber, we first estimated the uncontaminated δ^13^C of CO_2_ at the outlet of the chamber (δ^13^C_outlet pure_), by solving for δ^13^C_outlet_ the Eq. 10 of Evans et al. [34]

5 $$\begin{array}{l}\delta {\:^{13}}{{\rm{C}}_{{\rm{outlet}}\:{\rm{pure}}}}\: =\\ \:({\Delta ^{13}}{\rm{C}}\: + \:\xi \:\delta {\:^{13}}{{\rm{C}}_{{\rm{inlet}}}}\:({\Delta ^{13}}{\rm{C}}/1000)\: +\\ \:\:\xi \delta {\:^{13}}{{\rm{C}}_{{\rm{inlet}}}})\:/\:((\:{\Delta ^{13}}{\rm{C}}/1000)(\:\xi \: - \:1)\: + \:\xi ),\end{array}$$

with Δ^13^C given in per mil (‰). In Eq. 5, δ^13^C_inlet_ corresponds to the δ^13^C of CO_2_ as measured at the inlet of the growth chamber. Δ^13^C was set to 21‰, a value close to that estimated for shoot biomass of perennial ryegrass or temperate (C_3_) grassland in the absence of drought stress in many works [53–56]. ξ was obtained as [34]:

6 $$\xi=\:{C}_{\text{i}\text{n}\text{l}\text{e}\text{t}}\:/\:({C}_{\text{i}\text{n}\text{l}\text{e}\text{t}}\:\--\:{C}_{\text{o}\text{u}\text{t}\text{l}\text{e}\text{t}}),$$

with *C*_inlet_ and *C*_outlet_ the CO_2_ concentration in air as measured at the inlet and outlet of the growth chamber, respectively.

Next, we estimated δ^13^C_Ref_, the contamination-free δ^13^C representative for all functional parameters (biomass fractions, WSC components or dark respiration; see below) as,

7 $$\begin{array}{l}{{\delta\:}}^{13}{\text{C}}_{\text{R}\text{e}\text{f}}\:=\\\:({{\delta\:}}^{13}{\text{C}}_{\text{i}\text{n}\text{l}\text{e}\text{t}}\:\times\:{F}_{\text{i}\text{n}\text{l}\text{e}\text{t}}\:\--\\\:{{\delta\:}}^{13}{\text{C}}_{\text{o}\text{u}\text{t}\text{l}\text{e}\text{t}\:\text{p}\text{u}\text{r}\text{e}}\:\times\:{F}_{\text{o}\text{u}\text{t}\text{l}\text{e}\text{t}})\:/\:({F}_{\text{i}\text{n}\text{l}\text{e}\text{t}}\:\--\:{F}_{\text{o}\text{u}\text{t}\text{l}\text{e}\text{t}}).\end{array}$$

Then, *d*δ^13^C_Ref_, the uncontaminated δ^13^C-difference between δ^13^C_ref_ estimates for the parallel chambers, was obtained as the numerical difference between the two δ^13^C_Ref_ values. In the process, we used *d*δ^13^C_Ref_ in all calculations of *f*_contam_ for all types of samples and treatments, thus– for the time being– positing that Δ^13^C did not differ between treatments and that eventual post-photosynthetic discrimination was constant. In a second step, however, we explored the sensitivity of contamination estimates to variation of Δ^13^C during daytime gas exchange measurements, as observed in the different [CO_2_] treatments.

### Statistical analysis

One-way analysis of variance (ANOVA) with Tukey’s HSD post hoc tests for pairwise comparisons was conducted to explore the effect of CO_2_ treatments on the contamination (*f*_contam_) of biomass (*n* = 2–4) and WSC components (*n* = 2–4). For *f*_contam_ of dark respiration (*n* = 17–39), a linear mixed-effects model (LMM) was fitted using the lme4 package [57]. The model included [CO_2_] treatment as a fixed effect and sampling day as a random effect to account for temporal pseudo-replication. The significance of fixed effects was evaluated using sequential (Type I) likelihood ratio tests, and post hoc pairwise comparisons performed with Tukey’s HSD using the emmeans package [58]. All statistical analyses were performed in R v.4.0.2 [59]. The R-package ggplot2 [60] was used for data visualization.

## Results

### Variation of [CO_2_] and δ^13^C_CO2_ during the experiment

**Fig. 4:**
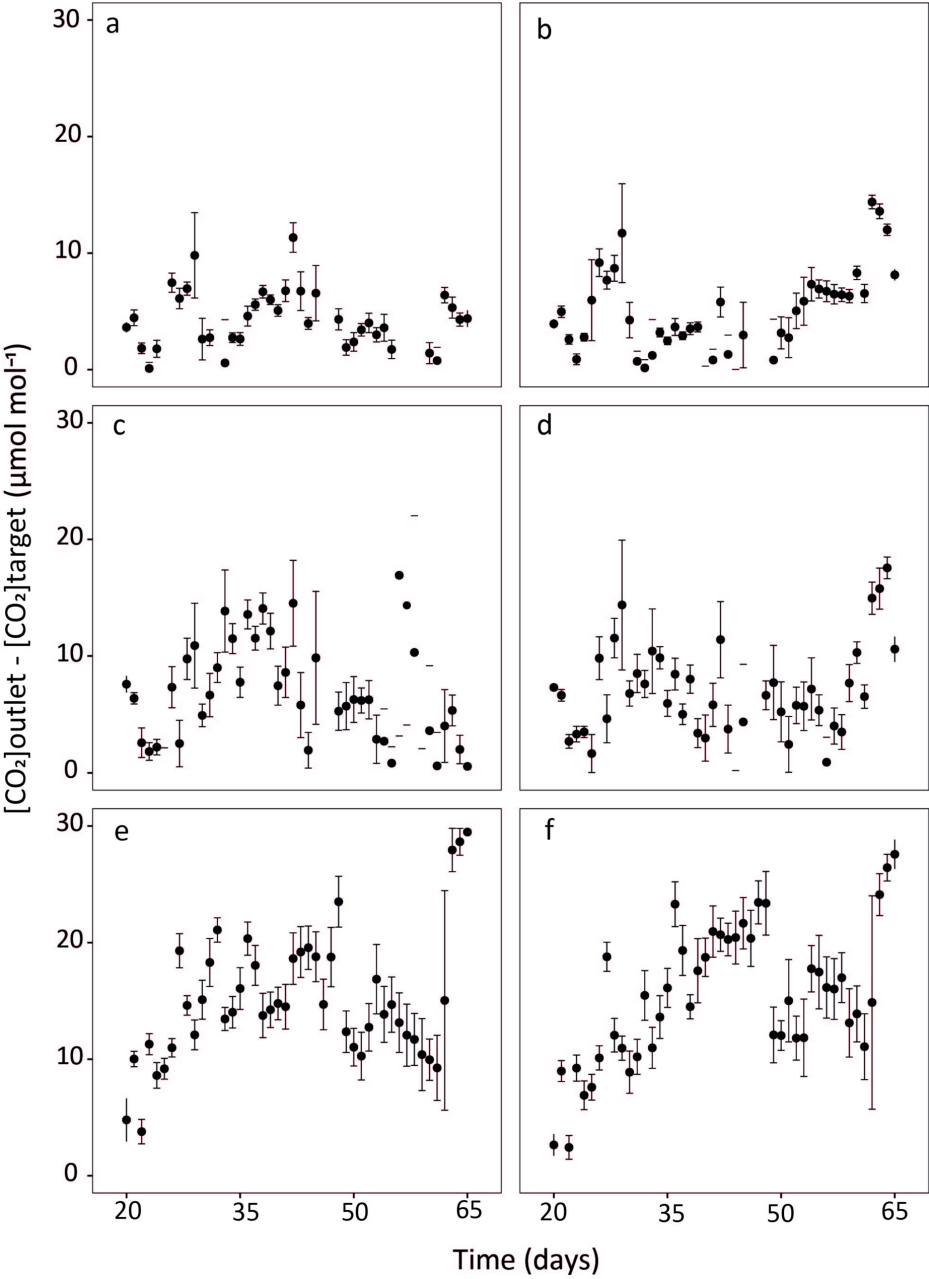
CO_2_ concentration difference between chamber outlet ([CO_2_]_outlet_) and the set target [CO_2_] ([CO_2_]_target_) between day 20 and 65 in experimental runs with target [CO_2_] of: (**a**, **b**) 200, (c, d) 400 and (e, f) 800 µmol mol^− 1^. Growth chambers were supplied with either ^13^C-depleted CO_2_ (δ^13^C_CO2_ -43.5‰) (panels **a**, **c** and **e**) or ^13^C-enriched CO_2_ (δ^13^C_CO2_ -5.6‰; right) (panels **b**, **d**, **f**). Measurements taken during the first 45 min of the light period, or following the opening of the chamber, or exceeding 1.5 times the interquartile range (outliers) were eliminated from the data set. Data points and error bars represent daily means ± SD (*n* = 9–23)

The daytime mean CO_2_ concentration at the chamber outlet varied little (coefficient of variation < 2%) between 20 and 65 days, and on average was 4.0 (±4.3 SD), 7.2 (±6.2 SD) and 13.9 (±8.3 SD) µmol mol^− 1^ higher than the target [CO_2_] of 200, 400 and 800 µmol mol^− 1^, respectively (Fig. 4). These differences corresponded to mean relative deviations from target [CO_2_] of ≤2% in every treatment. Importantly, these deviations did not differ (*P >* 0.05) between chambers receiving ^13^C-depleted and ^13^C-enriched CO_2_ (Fig. 4).

**Fig. 5:**
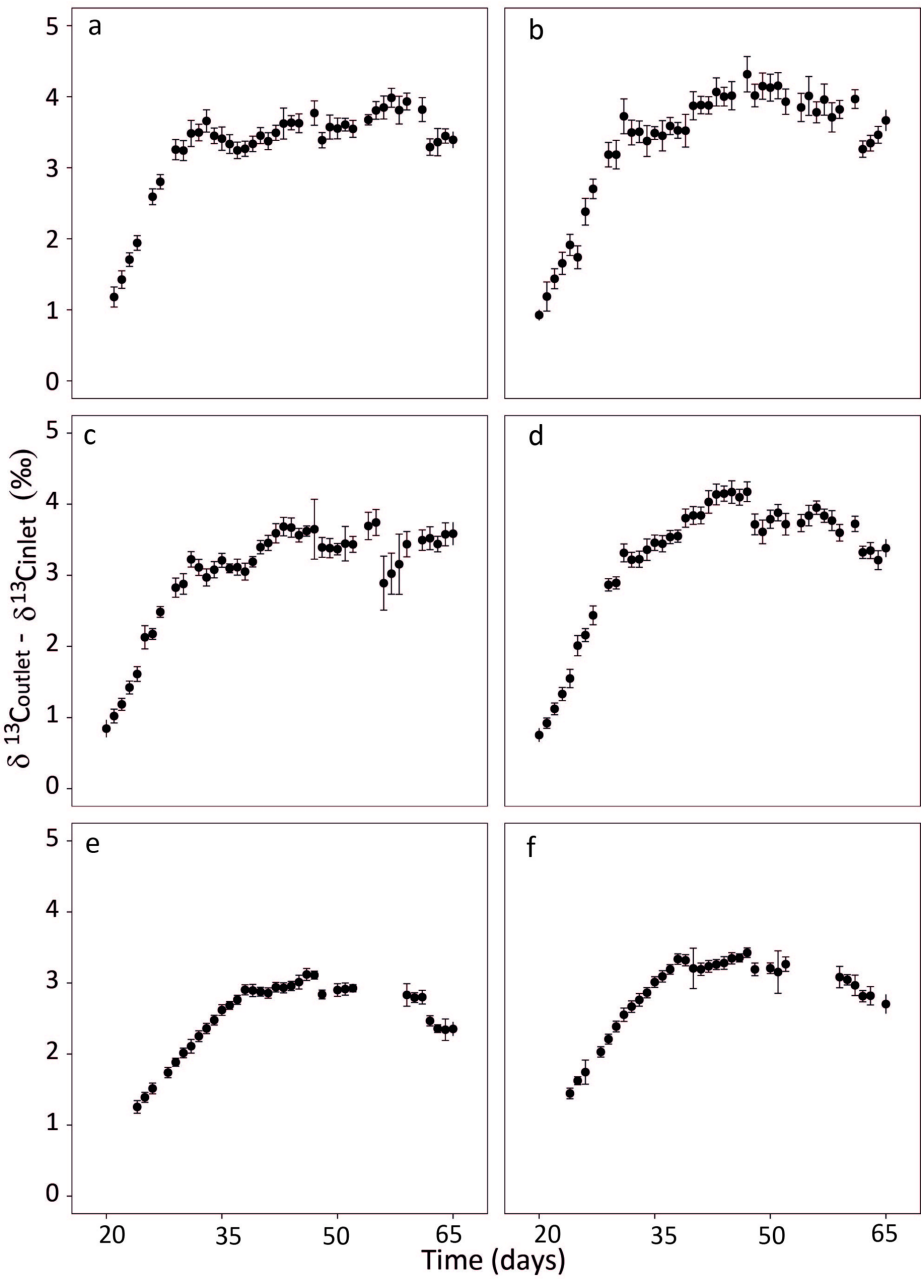
The δ^13^C-difference between CO_2_ measured at the chamber outlet (δ^13^C_CO2 outlet_) and inlet (δ^13^C_CO2 inlet_) over time (δ^13^C_CO2 outlet_ - δ^13^C_CO2 inlet_). CO_2_ concentration at chamber outlet ([CO_2_]_outlet_) was maintained near target [CO_2_]: 200 (**a**, **b**), 400 (**c**, **d**) and 800 (**e**, **f**) µmol mol^− 1^ (see Fig. 4). Growth chambers were supplied with either ^13^C-depleted (δ^13^C_CO2_ -43.5‰; panels **a**, **c** and **e**) or ^13^C-enriched CO_2_ (δ^13^C_CO2_ -5.6‰; b, d and f). Measurements taken during the first 45 min of the light period, or following the opening of the chamber, or exceeding 1.5 times the interquartile range (outliers) were eliminated from the data set. Data points and error bars represent daily means ± SD (*n* = 9–23)

Meanwhile, the δ^13^C of CO_2_ at the chamber outlet (δ^13^C_CO2 outlet_) relative to the chamber inlet (δ^13^C_CO2 inlet_) increased by several ‰ during daytime until day 30 to 35 (Fig. 5) when canopies became closed. Thereafter, the increase of δ^13^C at the chamber outlet relative to that at the inlet was relatively stable until the end of the experiments (Fig. 5). Again, these effects were the same in chambers receiving ^13^C-depleted and ^13^C-enriched CO_2_ (Fig. 5).

### Contamination

ANOVA provided no evidence for a significant effect of [CO_2_] treatments on the fraction of contaminating C (*f*_contam_) in any parameter of the study, except for respired CO_2_ (Table 2).

Biomass components (shoot and root), including WSC-free shoot biomass, and the different WSC fractions shared a very similar contamination of (on average) 3.3% (±0.9% SD), which was– moreover– close to that of respired CO_2_ at both 200 and 400 µmol mol^− 1^ CO_2_ (compare in Table 3), and did not differ significantly (*P* = 0.84) between the latter. Conversely *f*_contam_ of respired CO_2_ was slightly negative at 800µmol mol^− 1^ CO_2_, but not significantly different from zero, and significantly smaller than at 200 and 400 µmol mol^− 1^ CO_2_ (*P* < 0.05 for both comparisons). Significantly, the uncertainty for the individual estimates of contamination (represented by the SD) was not much smaller than the contamination estimates for most biomass and WSC parameters (average SD 2.3%) and corresponded to an average coefficient of variation CV = SD/mean of 67%.

**Table 2:** Significance (*P*-value) of [CO_2_] treatment effects on contamination (*f*_contam_) parameters

	CO_2_ effect significance
	(*P*-value)
Biomass components	
Shoot	0.787
Root	0.219
Water-soluble carbohydrates	
Fructan	0.374
Sucrose	0.972
Glucose	0.816
Fructose	0.759
WSC-free shoot biomass	0.358
Dark respiration	< 0.001

CO_2_ treatment effects were tested with one-way ANOVA for biomass and WSC components (*n* = 2–4) and a linear mixed model for dark respiration (*n* = 17–39).

**Table 3:** The fraction of contaminating C (*f*_contam_, %) in diverse sample types

Parameter	CO_2_ concentration (µmol mol^− 1^)		
	200	400	800
	*f*_contam_, %		
Biomass components			
Shoot	3.9 (0.2)	4.1 (2.3)	2.7 (2.8)
Root	4.0 (0.7)	4.6 (1.6)	2.0 (1.4)
Water-soluble carbohydrates			
Fructan	3.7 (0.7)	2.2 (1.8)	4.8 (2.9)
Sucrose	3.4 (4.4)	2.7 (3.0)	3.4 (5.1)
Glucose	3.1 (4.2)	4.8 (2.3)	3.3 (5.1)
Fructose	3.7 (3.3)	4.5 (1.4)	1.9 (7.6)
WSC-free shoot biomass	3.6 (0.3)	4.3 (1.8)	2.1 (1.1)
Dark respiration	3.5 (2.7)^a^	3.5 (4.5)^a^	-2.4 (5.2)^b^

*f*_contam_ was determined for canopy-scale dark respiration for days 38 to 65, and bulk shoot and root C, and fructan, sucrose, glucose and fructose extracted and purified from shoot biomass sampled at the beginning of the light period on day 65. In all experiments, growth chambers were maintained near target [CO_2_] of 200, 400 or 800 µmol mol^− 1^ using one of two CO_2_ sources, a relatively ^13^C-depleted (δ^13^C -43.5‰) or ^13^C-enriched source (δ^13^C -5.6‰). *f*_contam_ for dark respiration was determined during periods of steady-state gas exchange of chambers. That is, measurements in the first 45 min of a dark period or following the opening of the chamber were removed, and values over 1.5 × IQR (Interquartile Range) away from the mean were removed as outliers. Except for [CO_2_] and δ^13^C_CO2_, all conditions were kept the same in all chambers (see Materials and Methods). The means and standard deviations (SD) are presented for each treatment and were calculated based on daily replicates (*n* = 17–39) for dark respiration measurements or chamber-level replicates (*n* = 2–4) for all other parameters. Different superscript letters in the same row indicate a significant (*P* < 0.05) effect of [CO_2_] treatments.

### The effect of varying discrimination on estimates of contamination

**Fig. 6:**
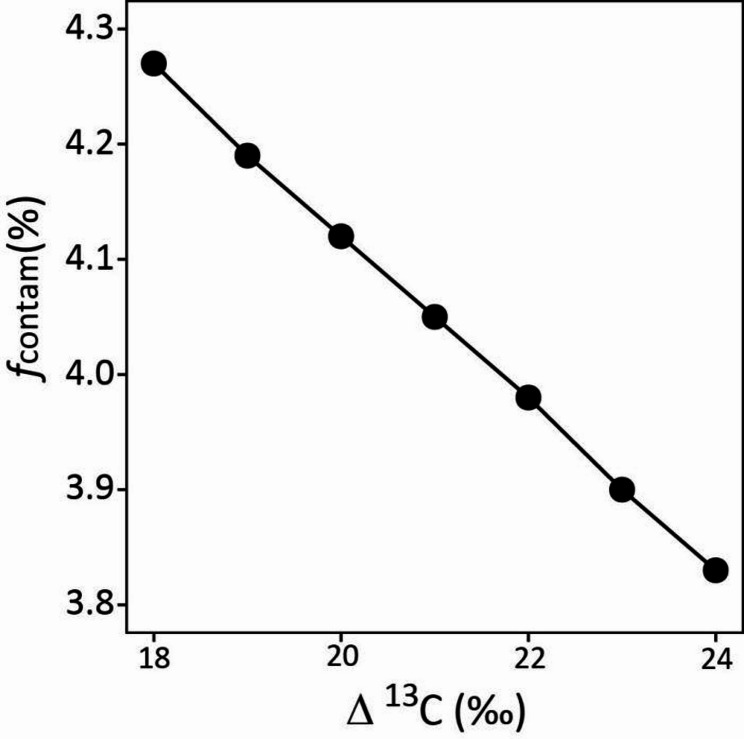
Sensitivity of contamination-% (*f*_contam_, %) estimates to assumptions of Δ^13^C in the range of 18 to 24‰. The analysis was based on an arbitrary sample with an estimated *f*_contam_ of 4.05% at Δ^13^C = 21‰ (see Materials and Methods)

As illustrated by the methodology, assumptions of Δ^13^C impact estimations of contamination (i.e. *f*_contam_) *via* the determination of the *d*δ^13^C_Ref_-values (see Eqs. 2, 5 and 7). Significantly, we observed [CO_2_] dependent variation of Δ^13^C during daytime net CO_2_ exchange (Δ^13^C_N_) counter to expectations: thus, Δ^13^C_N_ increased from approx. 19 to 23‰ between 200 and 800 µmol mol^− 1^ of CO_2_ (Table S1). Thus, our literature-based assumption of constant Δ^13^C (= 21‰) must have biased estimations of *f*_contam_ to some degree. The numerical effect of this Δ^13^C-dependent variation on estimates of *f*_contam_ is explored in Fig. 6.

This analysis demonstrated a negative relationship between estimates of *f*_contam_ and assumed Δ^13^C, with a 0.44% decrease of the estimated *f*_contam_ for a 6‰ decrease of Δ^13^C from 18 to 24‰. The maximum error on estimates of *f*_contam_ which resulted from neglecting the [CO_2_] treatment effect on Δ^13^C as observed here was 0.3%, but did not change conclusions with respect to the non-significance (or significance) of the [CO_2_] treatment effect on *f*_contam_ (Table S2).

## Discussion

### Contamination was small and similar for all parameters

To the best of our knowledge, this work presents the first systematic, comprehensive and quantitative assessment of isotopic contamination artifacts in a labelling experiment. This analysis determined a very small contamination of samples, which was– moreover– closely similar for a range of functional parameters (biomass fractions, WSC components and respired CO_2_) and not significantly different for the different [CO_2_] treatments (Tables 2 and 3), except for respiration at high CO_2_ which was insignificant. Lack of statistical significance for the [CO_2_] effect on contamination was not intuitive based on the expectation that incursion of a defined volume of extraneous CO_2_ into labelling vessels would cause a (proportionally) greater mixing with a low than a high set CO_2_ concentration, under *ceteris paribus* conditions. Indeed, there was a non-significant tendency for a lower contamination at 800 µmol mol^− 1^ [CO_2_] than at 200 and 400 µmol mol^− 1^, especially for the biomass components. Also, there was a significant (negative) [CO_2_] treatment-effect on *f*_contam_ for respired CO_2_, which accorded with the expected (relatively) smaller extraneous CO_2_ incursion at 800 µmol mol^− 1^ [CO_2_]. Yet, these effects were very small, and not even considering the [CO_2_] treatment-effect on Δ^13^C (Table S1) did change conclusions with respect to the (non-)significance of [CO_2_] treatment-effect on *f*_contam_ (Table S2). The fact that contamination was generally very small certainly contributed to the absence of statistical significance *via* a small signal to error ratio (which is– basically– the inverse of the CV) in the data. In that, the experimental error was not large at all (see also Materials and Methods). This may be recognized by translating a given contamination-% into the δ^13^C-difference (between ^13^C-enriched and ^13^C-depleted chambers), which is required to return a certain contamination-%. For instance, a 3% contamination corresponded to an approx. 1.1‰ smaller δ^13^C-difference between the measurements (*d*δ^13^C_X_) than the predicted uncontaminated reference estimates (*d*δ^13^C_Ref_). By comparison, with a very good average, whole-system SD of (say) 0.4‰ for the δ^13^C_X_ data– which integrates all errors from CO_2_ administration over an extended period of time, labelling chamber operation (including adjustments in flow rates, changes of CO_2_ flasks, variation of δ^13^C_CO2_ in the chambers, and sample collection and preparation)– error propagation yields a (whole system) SD of 0.57‰ on average for the *d*δ^13^C_X_ data. Given the average 1.1‰-signal associated with a 3% contamination (see above), this SD of 0.57‰ translates to a CV of 52% for the contamination estimate which is not far from that observed here for the biomass and WSC components (average 67%).

Clearly, increasing the isotopic spread between the two CO_2_ sources used in experiments would help to increase the signal-to-error ratio of contamination estimation. In our laboratory we have used commercial sources of CO_2_ with δ^13^C as high as −2‰ and as low as −50‰, which yields an isotopic spread which is somewhat larger than that found here (48‰ vs. 38‰). Of course, using artificially ^13^C-enriched CO_2_ sources [31] could further reduce the relative experimental error, including that of contamination estimations, and therefore increase to some degree the sensitivity of ^13^CO_2_/^12^CO_2_ tracer studies, albeit at much greater financial cost for the labelling CO_2_.

Importantly, in the present work contamination of the different WSC components was very similar to whole shoot biomass (from which they were extracted) and WSC-free shoot biomass. Based on this close similarity, we find no indication for any additional contamination which might have occurred during WSC extraction, separation and analysis. Given absence of evidence for additional contamination of WSC, it is futile to discuss any such eventual sources, except for acknowledging the effectiveness of the protocols and the cleanliness of the laboratory work.

Strikingly, contamination of respiratory CO_2_ at 200 and 400 µmol mol^− 1^ CO_2_ was also close to that of biomass and– specifically– WSC components. This observation agrees with the expectation that in vivo contamination of the respiratory substrate (specifically WSC) was the dominant factor explaining contamination of respired CO_2_ at least in these treatments. It is well accepted that non-structural carbohydrates are the dominant source of substrate for dark respiration [32, 61]. At the same time, this would also suggest that no additional contamination with extraneous CO_2_ occurred during respiration measurements. This is also unsurprising given the fact that dark respiration measurements occurred during (undisturbed) isotopic steady-state for gas exchange during periods when chambers had not been opened for at least 45 min previously. Meanwhile, we cannot explain the observation that respired CO_2_ was apparently uncontaminated at 800 µmol mol^− 1^ CO_2_, albeit this estimate was associated with relatively large uncertainty. Particularly, we have not found any chamber effects on any morpho-physiological parameters studied in the work of Baca Cabrera et al. [48–50], which occurred just prior to the tests which are presented here.

One question not directly explored by the present analysis is whether the δ^13^C_CO2_ of the contaminating source was more similar to the ^13^C-enriched or the ^13^C-depleted CO_2_ source used in this work. This question is also of interest for the accuracy of the Δ^13^C_X_ data which can be obtained from the present data. We opine that the actual δ^13^C_CO2_ of the extraneous (contaminating) CO_2_ was likely close to that of all CO_2_ exiting the chambers. Given that the fossil-organic and mineral CO_2_ sources were always used in parallel in equal proportions (see Materials and Methods) and CO_2_ was ^13^C-enriched by approx. 3‰ inside the chambers (see Fig. 5) due to photosynthetic ^13^C discrimination, we estimate the δ^13^C_CO2_ of this fifty-fifty mixture thus ≈ (0.5 × −43.5‰ + 0.5 × −5.6‰) + 3‰ = 27.6‰ (with −43.5‰ and −5.6‰, representing the δ^13^C_CO2_ of the fossil-organic and mineral CO_2_ supplied to the chambers). This δ^13^C-value of the total CO_2_ leaving the chambers is also close to the δ^13^C of human-exhaled CO_2_ (e.g. the experimenters) when this is based on a typical Central European, mainly C_3_-based diet [62]. Mixing of the CO_2_ inside the room housing the labelling facility (in the basement of ‘Alte Akademie 12’ in Freising-Weihenstephan) with free atmospheric CO_2_ (δ^13^C_CO2_ approx. − 9‰) was likely a very minor factor, as the volume of air in this room was continuously flushed with air from the growth chambers at a high rate. In consequence, we also argue that reasonable Δ^13^C_X_-values can be obtained by simply averaging the Δ^13^C_X_-values from the ^13^C-enriched and ^13^C-depleted chambers.

Although not comparable in terms of experimental purpose, system design and level of ^13^C enrichment, the degree of isotopic contamination observed in the present work seems comparable to that of commercial systems which are used to manufacture highly isotopically enriched compounds. Thus, for instance, closed systems [63] specially designed to produce highly isotopically enriched plant compounds with pure ^13^CO_2_ gas, achieved a degree of labelling of 96–98 atom-%. Given that isotopic fractionation is suppressed in a closed system [64] with continuous and complete photosynthetic fixation of the supplied substrate CO_2_, and also cannot occur for C when the added substrate CO_2_ contains only one C isotope (pure ^13^C in this case), it would seem that isotopic contamination (with ^12^C) in [63] was probably very similar at approx. 2–4%.

In the present work, contamination was likely dominated by extraneous CO_2_ entering the growth chambers during light periods when these had to be accessed for experimental or maintenance purposes (e.g. changes of defective light sources). Unfortunately, we did not sample the 12 days-old seedlings when we started the δ^13^C_CO2_ treatments, so we cannot quantify the possible contribution of the experimental starting material (see Background) to the integral contamination estimate. However, if we make assumptions extrapolated from our first chamber-scale gas exchange measurements, we estimate an experimental starting material-associated contamination of not more than ~ 1% (compare also plant sizes in Figure S4).

### How to deal with contamination in tracer data evaluation?

Of course, the best way to avoid complications with contamination is to avoid contamination altogether. As we emphasize, using air locks in chamber doors and minimizing experimental and maintenance operations inside the chambers during daytime are important contamination avoidance principles in addition to precautions already mentioned in the Discussion sections above. Concerning air locks, there may be a trade-off between their effectiveness in reducing CO_2_ incursion when doors are open and the ease of access to the chamber interior that they permit (compare Figures S3A and B). While we failed to compare the effectiveness of these two versions of air locks directly, the measurements by Lehmeier et al. [32] do suggest that their airlocks provide excellent proof for their effectiveness (Figure S3B).

The fact that we observed only small contamination, despite of the fact that the study was performed with a highly experimentally-perturbed system, supports our assessment that previous works which were performed with less experimentally disturbed studies in a very similar system [25, 32] should have suffered even less from contamination. This view is supported by the absence of a CO_2_ source (^13^C enriched vs. ^13^C-depleted CO_2_) effect on measurements of Δ^13^C during net CO_2_ exchange in light [25]. Nevertheless, for instance, Lehmeier et al. [32] did allow for some contamination in their evaluation of the tracer kinetics of respired CO_2_ when using a very similar, two-chamber system with two distinct δ^13^C_CO2_. In that, they used measurements from plants which had grown continuously in the presence of ^13^C-enriched or ^13^C-depleted CO_2_ as the endmembers (δ^13^C_new_ and δ^13^C_old_) of the isotopic mixing model which they applied to the tracer data. This procedure did correct for an eventual contamination, although it used the assumption that contamination was a constant.

## Conclusions

The aim of this work was to quantify systematically and comprehensively C isotopic contamination artefacts which occurred in a > 9 weeks-long experiment with continuous exposure of *L. perenne* plants to one of two C-isotopically distinct natural CO_2_ sources, one a ^13^C-depleted fossil-organic source and the other a (relatively) ^13^C enriched mineral source, at one of three [CO_2_]-levels: 200, 400 or 800 µmol mol^− 1^ CO_2_ in plant growth chambers. The experiments provided an elevated opportunity for contamination due to extensive experimental activities in all chambers during the last two weeks just prior to determination of contamination. Nevertheless, the findings indicated only a low level of contamination (3.3% on average) for biomass and WSC fractions, with no significant effect of [CO_2_] on contamination. Thus, our work supports the use of the present ^13^CO_2_/^12^CO_2_ system and protocols for quantitative C tracer experiments of plant metabolism across contrasts of [CO_2_]. Certainly, contamination avoidance principles used (and discussed) here should be adopted also in simpler tracer systems (e.g. one-chamber systems with or without inclusion of CF-IRMS or other online gas isotope analysers) in controlled or field environments [21, 31], especially if such experimental systems do not permit quantification of contamination artifacts, as is usually the case.

## Supplementary Information

Below is the link to the electronic supplementary material.

Supplementary Material 1

## Data Availability

The data supporting the findings of this study are available from the corresponding authors upon reasonable request.
