# Supplementary material for: Assessing and avoiding C isotopic contamination artefacts in mesocosm-scale 13CO2/12CO2 labelling systems: from biomass components to purified carbohydrates and dark respiration
Source: Plant Methods. 2025 Aug 11;21:111. doi: 10.1186/s13007-025-01431-3 (PMC12337381; doi:10.1186/s13007-025-01431-3)
Supplement: Supplementary file 1 — Supplementary Material 1 [file 13007_2025_1431_MOESM1_ESM.pdf]

Supplemental Information

Table S1 ^{13}C discrimination ($\Delta^{13}C$) during CO_2 exchange in light at different atmospheric CO_2 concentrations as determined in chambers supplied with ^{13}C-enriched or ^{13}C-depleted CO_2.

CO_2 concentration ($\mu mol\ mol^{-1}$)	$\Delta^{13}C$ (‰)	
	^{13}C-enriched CO_2	^{13}C-depleted CO_2
200	18.8	18.8
400	20.3	20.1
800	23.1	22.4

Different letters in the same column indicate a statistically significant effect at $P<0.05$. For details, see Materials and Methods.

Table S2 The fraction of contaminating C (f_{contam}, %) in diverse sample types.

Parameter	CO_2 concentration ($\mu mol\ mol^{-1}$)		
	200	400	800
		f_{contam} (%)	
Biomass components			
Shoot	3.9 (0.2)	4.1 (2.3)	2.6 (2.8)
Root	4.1 (0.6)	4.6 (1.6)	1.9 (1.3)
Water-soluble carbohydrates			
Fructan	3.8 (0.7)	2.2 (1.8)	4.7 (2.9)
Sucrose	3.5 (4.4)	2.8 (3.0)	3.3 (5.1)
Glucose	3.2 (4.2)	4.8 (2.2)	3.2 (5.1)
Fructose	3.8 (3.3)	4.6 (1.4)	1.8 (7.6)
Dark respiration	3.6 (2.7)[a]	3.6 (4.5)[a]	-2.5 (5.2)[b]

f_{contam} was determined for bulk shoot- and root-C, and water-soluble carbohydrate components (fructan, sucrose, glucose and fructose) extracted and purified from shoot biomass samples. Calculations were based on the average $\Delta^{13}C$ (Table S1) observed at the different [CO_2] levels. Different superscript letters in the same row indicate a significant (P < 0.05) effect of [CO_2] treatments. For further detail, see Table 2.

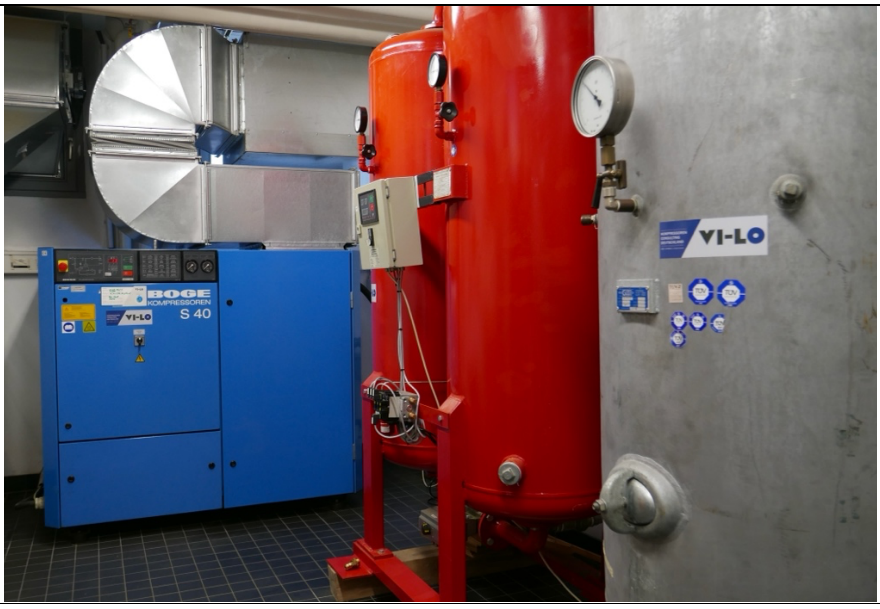

Figure S1 The main parts of the system generating a continuous supply of CO_2-free air. From left to right: air compressor (blue), self-regenerating adsorption dryer (red), and air receiver (grey). For technical detail, see main text.

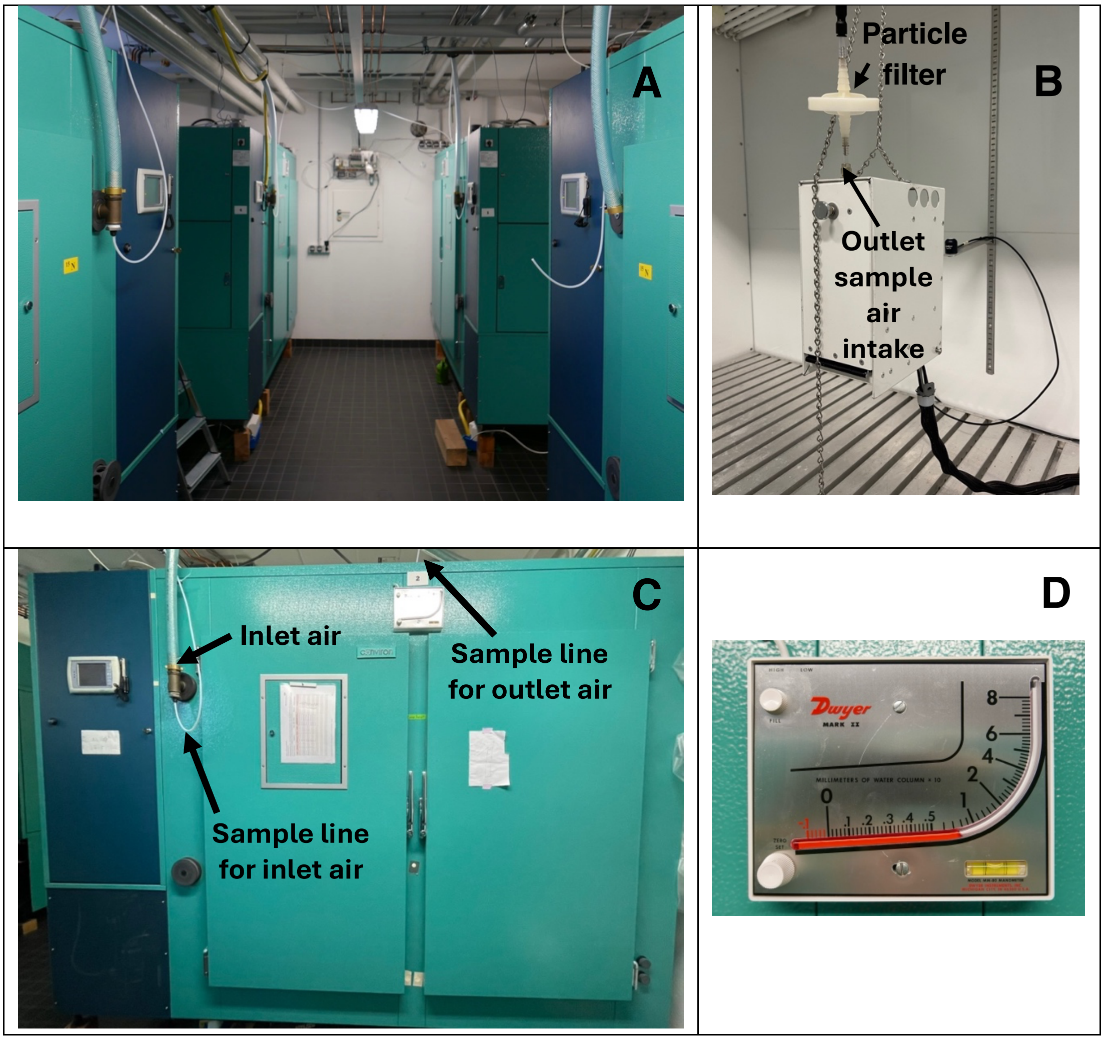

Figure S2 (A) Arrangement of the four plant growth chambers which form part of the $^{13}CO_2/^{12}CO_2$ gas exchange and labelling facility; (B) point of outlet air sampling intake inside the plant growth chamber, about halfway between the top of the plant canopy and the lamps; (C) plant growth chamber, with points of air inlet, and sample air lines at inlet and outlet. For detail, see main text; (D) pressure gauge of the plant growth chamber showing a slight overpressure inside the chamber relative to ambient atmospheric pressure. For more technical detail, see main text.

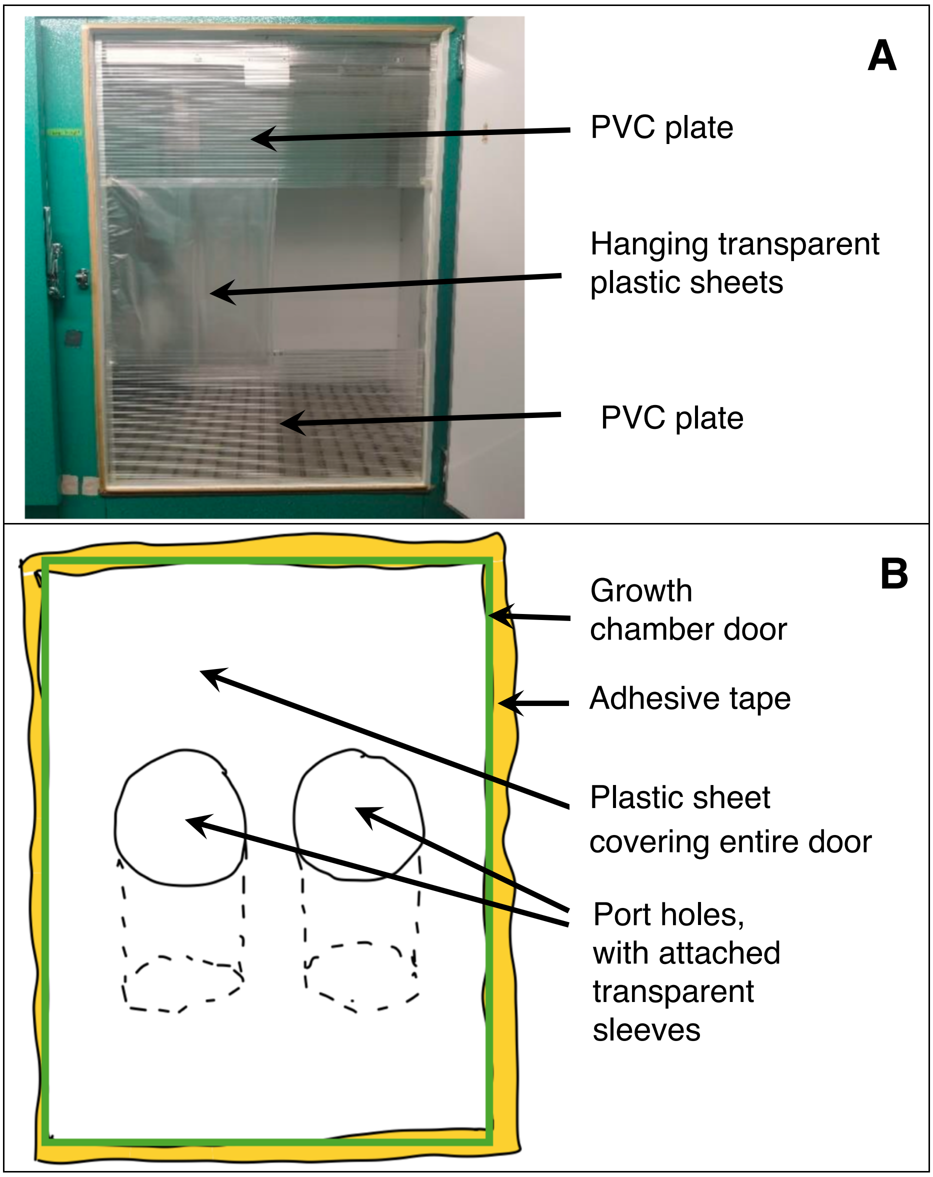

Figure S3 (A) Air lock as used in the present work, consisting of two PVC plates (770 × 460 mm) and two plastic sheets (570 × 400 mm). PVC plates covered the upper and lower parts of the door. The middle area was covered with two hanging plastic sheets to enable access to the chamber. (B) Sketch of air locks as used in Lehmeier et al. (2008): "*Chamber doors were equipped with custom-made transparent air locks that had small ports through which plants could be handled and sampled. […] Empty chamber tests of air locks demonstrated that with doors opened for 20 min, the CO_2 concentration in the chambers changed by only 4 $\mu L\ L^{-1}$ and $\delta^{13}C$ changed by about 1‰. Twenty minutes after closing the chambers, CO_2 concentration and $\delta^{13}C$ in the chambers had returned to set-point values.*"

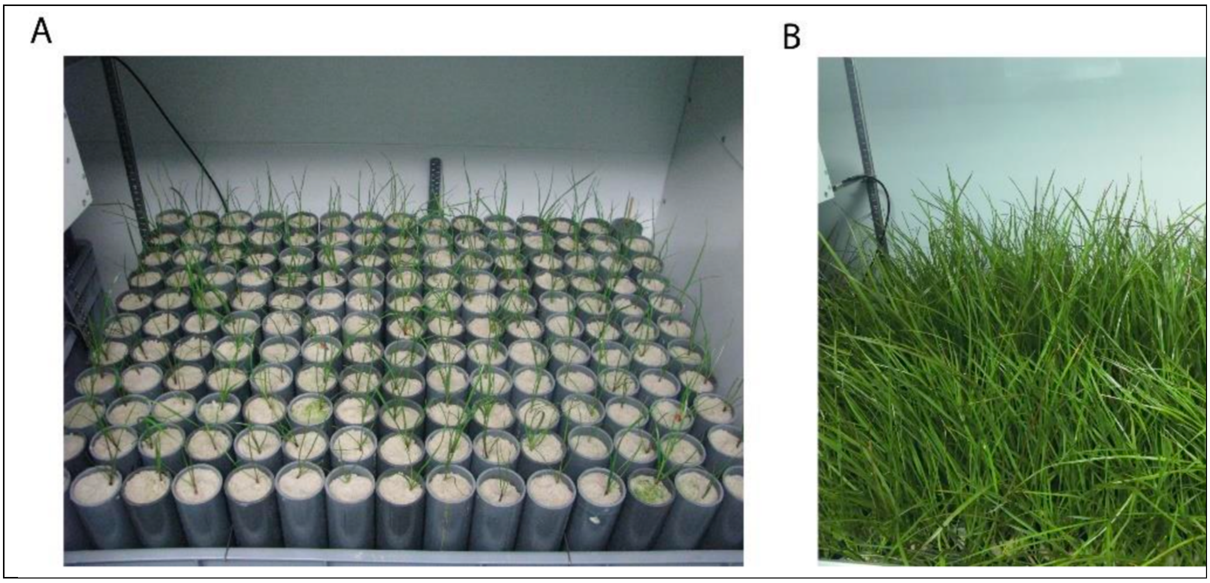

Figure S4 Typical stages of plant and stand development during the experiment: (A) arrangement of plants in the growth chamber at day 18 after imbibition of seeds, 6 days after the imposition of the [CO_2] treatments; (B) dense canopies had developed by day 49. Treatments did not exhibit differences in development rate or leaf dimensions (Baca et al., 2020). All canopies were closed with a leaf area index >5.5 at the time of individual plant sampling (Baca et al., 2020).

References

Baca Cabrera JC, Hirl RT, Zhu JJ, Schäufele R, Schnyder H. Atmospheric CO_2 and VPD alter the diel oscillation of leaf elongation in perennial ryegrass: compensation of hydraulic limitation by stored-growth. New Phytol. 2020;227:1776–89.

Lehmeier CA, Lattanzi FA, Schäufele R, Wild M, Schnyder H. Root and shoot respiration of perennial ryegrass are supplied by the same substrate pools: assessment by dynamic ^{13}C labelling and compartmental analysis of tracer kinetics. Plant Physiol. 2008;148:1148–58.
